# Prevalence and Predictor of Impostor Phenomenon Among Medical Students at Jouf University, Saudi Arabia

**DOI:** 10.7759/cureus.48866

**Published:** 2023-11-15

**Authors:** Marwa Elnaggar, Taif Alanazi, Norah A Alsayer, Maram Alrawili, Rahaf Alanazi, Ranyah Alghamdi, Roond Alrwili

**Affiliations:** 1 Medical Education, College of Medicine, Jouf University, Sakaka, SAU; 2 Medical Education, College of Medicine, Suez Canal University, Ismailia, EGY; 3 Community and Family Medicine, College of Medicine, Jouf University, Sakaka, SAU

**Keywords:** impostor phenomenon, imposter syndrome, academic performance, prevalence and predictor, medical students

## Abstract

Background and aim: The impostor phenomenon (IP) was defined in 1978 as an "internal feeling of intellectual phoniness felt by people with outstanding academic and professional achievements." This study aims to estimate the prevalence and predictors of IP among medical students.

Methods: A cross-sectional study was conducted from November 2022 to May 2023. A total of 200 medical students from years one to five participated in the survey; the response rate was 82.5% (165). A stratified random sampling technique was used to select groups of male and female Jouf University medical students from the 1st, 2nd, 3rd, 4th, and 5th batches of the 2022 to 2023 academic year. An e-mail with a self-administered questionnaire comprising the Clance IP scale and a consent form was sent to all participants. The identities of the students were kept anonymous to eliminate response bias. Participation was voluntary. Data were presented in terms of numbers and percentages; a chi-square test was used to compare categorical variables; and multivariable analysis was used to determine predictors of IP using SPSS Statistics version 20 (IBM Corp., Armonk, NY, USA).

Results: A total of 165 students responded to the questionnaire, with a response rate of 82.531%; 47.30% were males, and 86 (52.10%) were females. The findings indicate that 12 (7.3%), 83 (50.30%), 59 (35.80%), and 11 (6.70%) medical students, respectively, suffered from few, moderate, frequent, and intense imposter features. Results also showed that 13 (56.50%) first-year students suffered from moderate IP experiences, and 10 (43.50%) had frequent IP experiences. It was observed that for students in the first year, the academic performance represented by a student's grade point average (GPA), monthly family income, father's educational level, mother's educational level, and marital status are predictors of impostor experience.

Conclusion: The study's findings show that impostor syndrome is a common problem among medical students and that being in the first year of medical school can increase the incidence of suffering from it. The problem-based learning system in medical school can contribute to impostor syndrome among medical students. Both low-achieving and high-achieving students suffer from impostorism.

## Introduction

Medical students are subjected to a variety of pressures, including the pressure to live up to their families or their own expectations, high competencies, and the high-demand nature of medical schools. Medical school can be a tremendous shock for high-achievers who have achieved academic success, resulting in low self-esteem and self-doubt. This can lead to something called impostor feelings. American clinical psychologists Pauline Clance and Suzanne Imes defined the impostor phenomenon (IP) in 1978 as an "internal feeling of intellectual phoniness felt by people with outstanding academic and professional achievements". Initially, it was used to describe high-achieving women who believed they were less intelligent or capable than others perceived them to be. Since then, the IP has been observed and documented at similar levels for both genders [1]. The IP was also identified in high-achieving medical students; it's probably unsurprising that it affects so many of them. It is linked to elevated levels of stress, perfectionism, and low self-esteem, which all contribute to IP. [2] Imposter phenomena are also high during transitional phases from basic to clinical years, possibly due to a lack of experience, as stated in the study conducted by Bravata et al. [3].

When Clance and Imes first discussed IP in 1978, they described it as an incapacity to internalize success and a propensity to attribute achievement to uncontrollable variables like luck, mistakes, or having the proper connections. Impostorism, or the inability to acknowledge one's own or another's success, is a typical outcome of this shortcoming [4]. Students often face a steep learning curve as they transition into clinical clerkships, as they are now expected to integrate themselves into a medical team, learn clinically rather than didactically, clerk, examine, perform procedures on actual patients, and assist with various aspects of patient care. Students accidentally produce a sense of fraudulence by attempting to play the role of a young doctor while being conscious of their limitations. Finally, 'pimping' is a widespread practice in which senior medical students challenge junior medical students about clinical issues in front of the team or patients. While this usually benefits learning, 'malignant pimping,' done violently, at an unnecessarily loud volume, or intending to humiliate, can harm students' self-esteem [5].

The IP is characterized by the belief that one is a phony among equally talented coworkers and the denial of one's achievements. Abdelaal stated that anyone can experience IP; this includes students and scientists at the pinnacle of their academic careers [6]. Khan clarified that the field of medicine is one of achievement. Medical school is a hive of knowledge testing, day in and day out. The breadth and depth of medical knowledge are constantly expanding, and there is pressure on students and professionals to keep up. The practice of medicine fosters imposter syndrome [7].

Ng and Tay found that the prevalence of low self-esteem and positive imposter syndrome was 23.6% and 42.1% (n = 573), respectively. There is a positive association between low self-esteem and positive imposter syndrome. Significant associations exist between self-esteem and gender, maternal education, and grade point average (GPA). The IP is significantly associated with gender, as stated by Alsaleem et al. [8].

Most of the literature suggests that medical students have a high prevalence rate of IP; even though IP was initially described over 40 years ago, the predictors of suffering from it and its effect on medical students’ academic performance are covered in a few studies. Most research on IP focuses on medical students; this may not be shocking considering that medical school is a crucial period for physician-in-personal training and professional growth. Knowing that IP tends to repeat during transitional periods makes it helpful to study medical students, particularly during these phases. Self-doubt and poor self-esteem can grow in medical school without direction and support, predisposing learners to develop IP later in their training and profession [9].

This study aimed to measure the prevalence and predictors of IP among medical students at the College of Medicine, Jouf University, Saudi Arabia. The objectives were to evaluate the prevalence of IP, identify factors contributing to IP among the study population, and compare the prevalence of IP among male and female students as well as basic science and clinical phase students.

## Materials and methods

Study setting and participants

This study is designed as a quantitative analytical cross-sectional study conducted in the College of Medicine, Jouf University, Saudi Arabia, between November 2022 and May 2023. The study included male and female medical students during their 1st, 2nd, 3rd, 4th, and 5th years of medical school studying during the academic year 2022-2023. They were stratified and randomly selected. Medical students from other universities besides Jouf University and health science students at Jouf University were excluded. A self-administered questionnaire comprising the Clance IP scale collected details on sociodemographic characteristics and academic performance.

The population was divided into groups (strata) first by batch (1st, 2nd, 3rd, 4th, and 5th-year batches), then by gender (male and female), according to a stratified random sampling design. A straightforward random sample (stratum) was taken from each group, with 40 students in each stratum. We gathered information on each stratum (sampling unit) that was chosen at random from each group. We selected a homogeneous stratum. The questionnaire was constructed and distributed via Google Forms (Alphabet Inc., Mountain View, CA, USA), and students had to register via university official emails. An email containing the questionnaire and informed consent forms was sent to participants in all groups. The identities of the students were kept anonymous to eliminate response bias. The sample size was estimated. Using the single proportion sample size calculation formula, a sample size of 200 was required, with a confidence level of 95% and a precision of 5%.

The Clance IP scale was created to assess the idea that people who appear to be competent to others may not be successful in their own eyes. The scale evaluates the IP's various aspects. The Clance IP scale is a 5-point scale with response options ranging from 5 (strongly agree) to 1 (strongly disagree) [4]. No pilot study was needed in this research since the instrument used is a validated scale. It has been used in other cross-sectional studies regarding IP with similar populations.

The impostor rest (Clance IP scale) was designed to help determine if an individual has IP characteristics and, if so, to what extent they suffer. We summed up the number of responses to each statement after the impostor test (Clance IP scale) was taken. Respondents with a total score of 40 or less have few IP characteristics. A score of 41 to 60 indicates that the respondent has moderate IP experience. A score of 61 to 80 means that respondents frequently have impostor feelings. A score above 80 means that the respondent has intense IP experience. A higher score indicates a more frequent and serious impostor phenomenon that interferes with a person's life.

Ethical consideration

Approval was obtained from the Jouf University Ethics Committee (approval no. 1-03-44). There weren’t any invasive methods used in data collection. The data collection tools used were anonymous. Informed consent was obtained from all participants.

Statistical analysis

Data were encoded, input, and processed using SPSS Statistics version 20 (IBM Corp., Armonk, NY, USA). A p-value < 0.05 was considered the cut-off value for statistical significance. Descriptive data, such as the distribution of gender and the distribution of the population over the years, were presented by number and frequency. Bivariate statistical analysis was performed using the appropriate chi-square test based on the study type and outcome variable. A p-value < 0.05 and 95% confidence intervals were used to demonstrate the statistical significance and precision of the results. Comparative statistics compared the statistically significant differences between male and female students. Multivariable analysis was used to determine predictors of IP for students of the basic sciences and clinical sciences phases [10].

## Results

Table 1 shows the demographic data of the study population; 86 (52.10%) were females, and 78 (47.30%) were males. Of the participants, 159 (96.4%) were aged between 18 and 24 years. Most were in the 2nd (57, 34.50%) and 3rd years (41, 24.80%). The GPA was more than 4.50 for 67 (40.6%). Regarding monthly income, 49 (29.70%) have a family income of more than 20,000 Saudi riyals (SR). A bachelor's degree was the most common for both the father’s (81, 49.1%) and mother’s (76, 46.10%) educational levels. Students with a GPA of >4.5 responded the most (67, 40.6%). Of the participants, 159 (96.40%) were single.

**Table 1:** Demographic data of the study population GPA: Grade point average, SR: Saudi riyal

Parameters	Categories	N (total n = 165)	Percentage
Sex	Male	78	47.3
	Female	86	52.1
	Prefer not to say	1	0.6
Age range	18-24 years	159	96.4
	25-30 years	6	3.6
	31-36 years	—	—
Academic year	1st	23	13.9
	2nd	57	34.5
	3rd	41	24.8
	4th	32	19.4
	5th	12	7.3
GPA	<3.00	7	4.2
	3.00-3.49	13	7.9
	3.50-3.99	36	21.8
	4.00-4.49	42	25.5
	4.50	67	40.6
Monthly family income (in SR)	<5000	31	18.8
	5000-10,000	22	13.3
	10,000-20,000	63	38.2
	>20,000	49	29.7
Father's educational level	Illiterate and elementary	19	11.5
	High school	29	17.6
	Diploma	16	9.7
	Bachelor's degree	81	49.1
	Postgraduate degree	20	12.1
Mother's educational level	Illiterate and elementary	28	17.0
	High school	23	13.9
	Diploma	32	19.4
	Bachelor's degree	76	46.1
	Postgraduate degree	6	3.6
Marital status	Married	5	3.0
	Single	159	96.4
	Widowed	1	0.6
	Divorced	0	0

**Figure 1:**
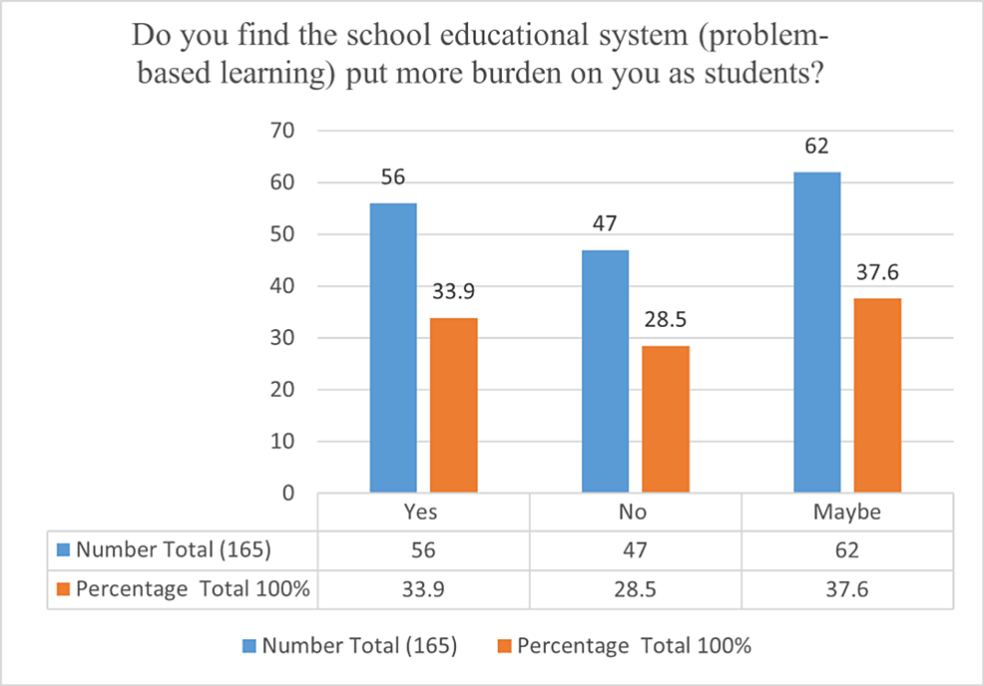
Medical students’ opinions on problem-based learning system

Figure 1 shows that 62 (37.60%) students found that the school educational system (problem-based learning) puts more burden on them.

**Figure 2:**
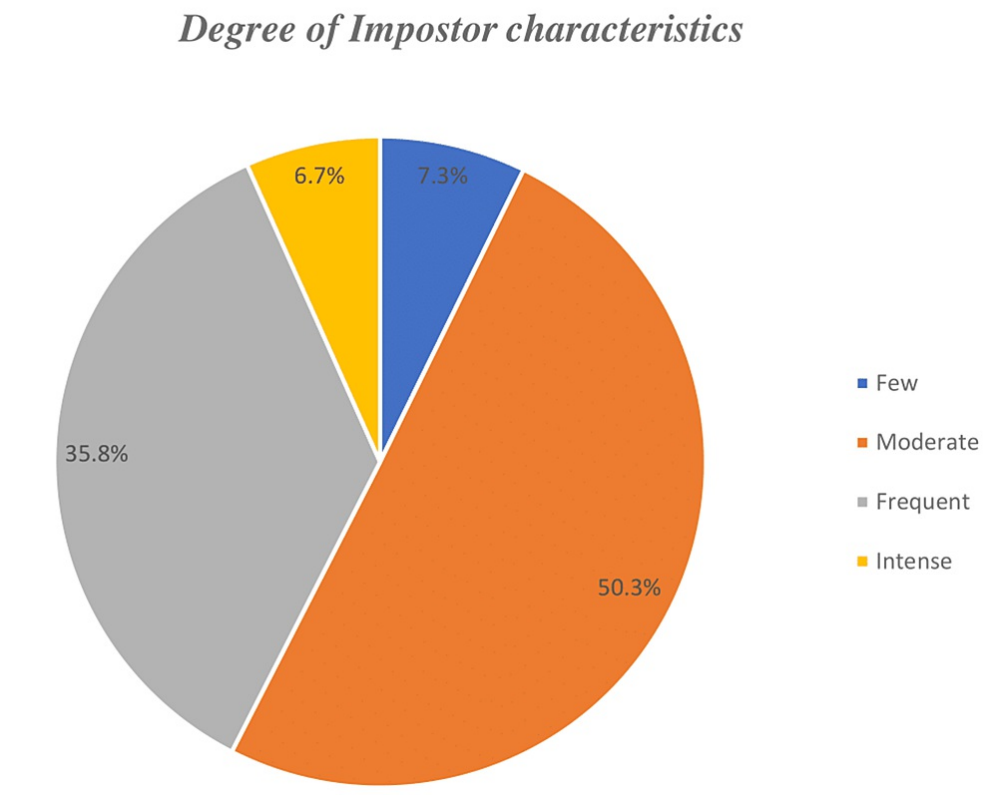
Degree of impostor characteristics among medical students at Jouf University

Figure 2 shows the degree of impostor characteristics among medical students: 12 (7.3%), 83 (50.30%), 58 (35.80%), and 11 (6.70%) were suffering from few, moderate, frequent, and intense impostor characteristics, respectively.

Table 2 shows that almost half of the participants (83, 50.30%) had moderate IP experiences; it was nearly equal between males and females (40 and 43, respectively), and 58 (35.40%) had frequent IP experiences, which was roughly similar between males and females (30 and 28, respectively). An almost identical number of students had few (12, 7.30%) and intense (11, 6.70%) IP experiences, which was more in females. There were no statistically significant differences between males and females with respect to impostor characteristics (p ≥ 0.5).

**Table 2:** Degree of impostor characteristics among male and female medical students at Jouf University IP: Impostor phenomenon

Gender	Few impostor characteristics		Moderate IP experiences		Frequent IP experience		Intense IP experience		p
	≤ 40		between 41-60		61-80		≥ 80		
	N	%	N	%	N	%	N	%	0.584
		Male		4						5.10	40	51.30	30	38.50	4	5.10
Female	8	9.30	43	50.00	28	32.60	7	8.1	
Total	12	7.3	83	50.30	58	35.40	11	6.70	

According to Table 3, the majority of 1st year participants exhibited few, and intense impostor traits, whereas the 5th year participants exhibited no intense impostor traits. Almost half the students in all the academic years had moderate impostor experiences, ranging from 42.10% in the 2nd year to 59.40% in the 4th year. There were no statistically significant differences between students of the basic sciences and clinical sciences phases across the academic years with respect to impostor characteristics (p ≥ 0.5).

**Table 3:** Degree of impostor characteristics among different academic years of students in the basic and clinical science phases P < 0.05 shows statistically significant differences IP: Impostor phenomenon

Academic phase	Academic year	Few impostor characteristics		Moderate IP experiences		Frequent IP experience		Intense IP experience		p
		≤ 40		Between 41-60		61-80		≥80		
		N	%	N	%	N	%	N	%	0.600
Basic sciences	1st	0	0	13	56.50	10	43.50	0	0	
	2nd	5	8.80	24	42.10	24	42.10	4	7.00	
	3rd	4	9.80	20	48.80	12	29.30	5	12.20	
Clinical sciences	4th	2	6.30	19	59.40	9	28.10	2	6.30	
	5th	1	8.30	7	58.30	4	33.30	0	0	
	Total	12	7.3	83	50.30	59	35.80	11	6.70	

Table 4 shows that the following are predictors of having impostor experiences: studying in the first year, academic performance represented by GPA, monthly family income (SR), father's and mother's educational level, and marital status.

**Table 4:** Predictors of IP among medical students at Jouf University *p <0.05 show statistical significance differences IP: Impostor phenomenon, SR: Saudi royal, GPA: Grade point average

Predictors of IP	Adjustable odds ratio (AOR)	95% Confidence interval	p
Academic year (1st year)	1.769	0.504-6.207	0.373*
GPA less than 3	47.783	1.246-1831.98	0.038*
GPA from 3-3.49	27.793	0.969-797.311	0.052*
GPA from 3.50-3.99	26.125	0.859-794.741	0.061*
GPA from 4-4.49	15.975	0.534-478.068	0.110*
Monthly family income (SR) <5000	2.109	0461-9.655	0.336*
5000-10.000	4.218	1.158-15.361	0.029*
10.000-20.00	5.117	1.158-15.361	0.017*
Father's educational level: llliterate or elementary	1.949	0.433-8.766	0.384*
High school	0.224	0.034-1.460	0.118*
Diploma	0.248	0.053-1.090	0.065*
Bachelor's degree	0.254	0.040-1.599	0.144*
Mother's educational level: Illiterate or elementary	0.411	0.079-2.130	0.290*
High school	1.632	0.414-6.435	0.484*
Marital status: Married	0.067	0.005-0.914	0.043*

## Discussion

The authors aimed to estimate the prevalence and predictors of IP among medical students at Jouf University. We used the Clance IP scale, used in most investigations, which reported IP rates between 22% and 60%. In our study, an important finding is that males and females suffer from impostor phenomena of different intensities. While women do suffer from IP, half of the included studies that reported evaluating a gender effect found no difference in prevalence between males and females [3]. A study conducted by Russell et al. measured imposter syndrome in relation to gender across osteopathic medical schools and found that females reported higher levels of impostor syndrome than males [11]. Our study did not find gender-specific differences in the IP, and there was no statistically significant difference between males and females suffering from the IP. A study of 209 individuals found that women report higher impostor feelings than men in many studies, possibly due to lower self-esteem and higher neuroticism [12]. A study found that male impostors react more negatively to performance cues, such as negative feedback and perceived accountability, than female impostors [13].

Our study results show that 50.30% of medical college students suffer from moderate IP experiences, consistent with Alrayyes et al.'s study, which found that IP is highly prevalent among young adults in Saudi Arabia. Approximately 57.8% of the participating young adults were found to be suffering from IP [1]. One study found that up to 87% of an incoming class of medical students reported a high or very high degree of impostor syndrome [14]. The systematic review conducted by Bravata et al. found that the prevalence of impostor syndrome varies widely from 9% to 82%, largely depending on the recruitment strategy for the study [3].

A growing body of literature has investigated the differences in IP between medical students in the basic sciences phase and those in the clinical phase. Our study results showed that IP is more prominent in the basic sciences. A study of American medical students found that some students had surprisingly high levels of IP before matriculation and that the IP increased over the first year of medical school. [15] Another study of American medical students found that 65.4% of the medical students were found to be in the "clinically significant" impostor category [16], as they are on the verge of entering a highly demanding, practical life involving human lives.

A study by Levant et al. found that after at least one required eight-week clinical rotation has been completed, the transitional and early phases of clinical training begin. This is a noticeably stressful time, and a decline in confidence is linked to IP. In this study, third-year medical students reported mild to severe feelings of IP. In this study, third-year medical students reported moderate to intense feelings of IP [17].

Overall, the studies suggest that IP is prevalent among medical students and that there may be some gender differences in prevalence and reactions to certain cues. However, more research is needed to fully understand the differences between medical students in the clinical phase and the basic sciences phase [14-17].

The problem-based learning system implemented in the first three basic sciences years at the College of Medicine, Jouf University, is highly demanding on medical students and can increase students' suffering from IP as they shift from a spoon-fed learning method to self-directed learning. Despite their apparent competence, medical students secretly worry that their knowledge and talents fall short [14]. This secret will be open to others anytime during the problem-based learning sessions, especially brainstorming sessions. This study did not examine whether participants had impostor thoughts before entering the medical program or whether those feelings evolved during medical school. A study conducted by Legassie et al. concluded that moving on to senior classes or senior training years has no impact on IP feelings; therefore, if a student has the condition, they will continue to do so despite an increase in education level unless specifically treated using a tailored and customized approach [18].

Overall, the problem-based learning system in medical school can contribute to impostor syndrome among medical students. However, there are interventions that can be implemented to address this issue and improve the wellness of medical students, so numerous programs for the administration, personnel, and professors should be created. Students should receive training to help them recognize these students' needs and provide them with the assistance they need to deal with the issue. Workshops on mentoring, developing stress-relieving activities, selecting mentors, stating institutional requirements clearly, finding ways to improve confidence, and giving peer and workplace social support groups are some of the interventions. One study investigated the correlates of impostor syndrome in a medical education cohort and determined if an interactive workshop could improve wellness and defeat impostor syndrome. The study found that an interactive workshop can be effective in defeating impostor syndrome in medical education [19].

Regarding the predictors of the impostor phenomenon among medical students at Jouf University, our study found that being in the first year makes you suffer more compared with other years. The first year of medical school can be particularly challenging, as students are adjusting to a new environment and a new way of learning [20,21] in contrast with a study conducted by Zeb et al., who reported that the training year had no bearing on the impostorism grades [22]. With a GPA of less than 3/5 out of 5 and ranging from 3/5 to 4.49/5, students suffer more, which means that both low-achieving and high-achieving students are suffering more. A study of high-achieving medical students found that up to 87% of an incoming class reported a high or very high degree of IP [14]. It is possible to teach students about perfectionism and how to set more realistic and doable goals for themselves [23].

Monthly family income, father's and mother’s educational level, and marriage affect students suffering from IP. Several studies have investigated the predictors of IP among medical students. A study of Malaysian medical students found that IP was positively correlated with low self-esteem, depression, and anxiety [24]. Another study of international medical students found that IP traits were associated with poor professional performance [21].

Cokley et al. suggest that normalizing the experience through awareness and open dialogue between staff and managers, mentors, and preceptors will highlight its negative impacts and allow people to develop coping mechanisms [25]. As Heslop et al. concluded, anyone can experience imposter syndrome, although women, minorities that are underrepresented, trainees, and faculty in their early careers are particularly affected [26].

Additionally, some authors cautioned against generalized feedback because it might make IP sufferers feel even worse. The research has revealed helpful mitigation measures that work best when applied to individual approaches; a supportive, welcoming, and appropriate learning environment and workplace are essential for personal growth and job satisfaction. Imposter syndrome and stereotype threats must be addressed to reduce burnout and stress [27]. Caution is advised when developing time management strategies for people with IP to prevent exacerbating feelings of impostorism.

Dorsey suggested that awareness of the self-doubt cycle is helpful; understanding imposter syndrome does little to end it. Instead, one needs to take action. Medical students should use the skills they've developed by facing cultural challenges. This, Dorsey states, would permit them to experiment with new concepts and habits while undertaking new professional tasks and roles [27].

Limitations

The cross-sectional design of this study, which relied on self-reported data from a single class at a single medical school with an 82.50% response rate, has limitations. The results might not apply to different periods, learning institutions, curricula, or student types. The characteristics of respondents may be different from those of those who declined to participate in the survey. This observational study methodology cannot demonstrate causal linkages. There was a low response rate from 4th and 5th-year students; a solution for this would be to conduct interviews with the participants or use paper-based surveys. Another limitation was that our study lacked a psychological aspect. Psychological illness may be significant to the outcome, and previous studies have included mental illness and its effect on IP. The sample size of future studies might consist of all medical students in Saudi Arabia and compare the results of medical students from other institutions and students of other specialties at Jouf University.

We recommend that medical students understand IP. Awareness campaigns should target medical students on coping with impostor feelings. A greater understanding of the impostor predictors and the factors that effect suffering may foster student wellness and enhance experience as students transition from their preclinical to clinical phases of training.

## Conclusions

The study's findings show that impostor syndrome is a common problem among medical students and that being in the first year of medical school can increase the incidence of suffering from it. The problem-based learning system in medical school can contribute to impostor syndrome among medical students. Both low-achieving and high-achieving students suffer from impostorism.
